# Annual Address of the President of the Virginia State Dental Association

**Published:** 1878-02

**Authors:** 


### AMERICAN JOURNAL

OF

### DENTAL SCIENCE.

Vol. XI. THIRD SERIES—FEBRUARY, 1878. No. 10.

### ARTICLE I.

### *Annual Address of the President of the Virginia State Dental Association.*

[Delivered at the Eighth Annual Meeting of the Association, at Richmond, Va., Dec. 11th, 1877.]

GENTLEMEN OF THE VIRGINIA STATE DENTAL ASSOCIATION.—In obedience to that usage which demands of your President an Annual Address, we ask your attention to a few plain and practical reflections upon the profession of Dental Surgery in its legal aspects and relations in the State of Virginia.

Perhaps, we might more properly and accurately say, to the *absence* of all legal relation, recognition, protection or encouragement, under which the dentists of this Old Commonwealth have up to the present time, studied, cultivated and practiced a profession indispensable to the comfort and welfare of society.

It is, Gentlemen, a curious and inexplicable feature in Virginia legislation that almost every subject and interest and vocation, in which men occupy or employ themselves for their own, and for the public benefit should have received the fostering care and encouragement, the notice and protection of our laws—save and except the great and important subjects and interests of Medicine and Surgery.

From the preservation of sparrows and singing-birds, to the subsidy and endowment of Rail Roads, Canals and Universities; from the hatching of fish-spawn to the protection of our property, our character and our lives, the Codes and Statute books teem with enactments and provisions. The *ægis* of the law spreads its broad and ample folds over every subject and every interest, however trivial or however vital and important. Our affluence of law has passed into a proverb; our wealth of jurists, statesmen, philosophers and scholars has been our boast for years and years; and yet a great and important interest, inseparably identified with the health, the comfort and the happiness of society has been overlooked, neglected, or designedly remitted to the unaided care, and unrequited labor of the physicians and the surgeons.

With the exception of a few vague, and for the most part ineffectual provisions against malpractice, and the dispensing of poisons, "*Silent Leges*" might be written against medicine and surgery. The wise and the foolish, the cultured and the ignorant, the skilful and the blundering, the faithful and the conscientious, all occupy the same legal plane, and like our "American Citizens of African descent," are all "peers and equals" before *Virginia Law*, having the same rights in practice, enjoying the same privileges and immunities in administering to the professional needs of society, of relieving or aggravating human suffering, of restoring or maiming, curing or killing as accident, or good or ill fortune, may provide the service of a true physician and surgeon or the ministrations of the charlatan and impostor.

We repeat, that it is wonderful and inexplicable that uch interests involving the issues of human comfort and human life itself, should up to the present day have received so little attention, and awakened so little concern in the minds of our citizens, and their representatives in our General Assemblies; and equally amazing that this indifference and neglect should so long have been acquiesced in and submitted to by the legitimate representatives of medicine and surgery.

It is true, that recently the physicians of the State under the auspices of the Medical Association of Virginia have made a move in the right direction, and have asked at the hands of our legislators and lawgivers some recognition of the claims and needs of medicine and surgery and that the practice of these departments of science in Virginia may hereafter be regulated and controlled by judicious legal enactments and statutes. But we are informed by Professor Cabell, the President of the Medical Association, in his late address before that body, that nothing has been accomplished. A great and important measure "hangs fire" in our legislature from some unexplained cause. Perhaps it has not been pressed with sufficient energy, perhaps our representatives hesitate, because the subject has not been sufficiently popularized or sufficiently discussed and ventilated to impress them with a just sense of its importance and magnitude.

However that may be, the sad truth confronts us, that medicine and surgery, physicians and dentists, remain in "*Statu quo*," unrecognized, unprotected and unaided by any legal enactments or wholesome regulations for the practice of these important and indispensable professions.

And now, gentlemen, it becomes us, as the representatives of a cognate and intimately allied department of general science; suffering as we do the same disabilities, experiencing the same disadvantages, and laboring under the same and even greater obstructions to satisfactory, to successful development and compensating practice, and to the attainment of that *status* and consideration which are no less the object of a praiseworthy ambition, than the great and lasting benefit of our fellow men, that we "speak out," that we let our voices be heard, and our influence felt, in this crisis of our professional history.

Nay sirs, we are *more* urgently and imperatively called upon to take this position, and make this demand for laws regulating the practice of dental surgery in Virginia than are our friends the physicians and general surgeons. The difficulties we as dentists have to encounter, the wrongs and abuses we have to combat and contend against, are as flagrant, and as damaging as any that confront the physician or the general surgeon and if these, with all their traditional, established and time honored claims, privileges and advantages, experience the need of legal interposition and protection in the practice of their specialties of science, how much more urgent, and pressing is that necessity with *us*, whose relations, positions and *status* are yet even to-day subjects of debate, of contest, and of conflict.

We know, Sirs, it will be objected that we are asking "class legislation," that we are infringing, or attempting to infract the rights of others, for our own advantage and benefit. We shall be told, that all such matters and subjects should be allowed to adjust themselves, or to be arranged and settled by the fiat of public and popular opinion, etc. Such objections and arguments, while specious and plausible, are really unsound and untenable; and cannot bear the test of just criticism or fair analysis.

It cannot be "class legislation," because it is a question involving the interests and welfare of society, the comfort and safety of the patient, as well as the reputation and reward of the practitioners. In the "case at bar," as our friends the lawyers would phrase it, society is wholly and entirely comprised in the two classes, practitioners and patients, dentists and people; one, or the other or both, are benefitted or damaged, according to the character and capacity of our dentists, or the nature and quality of the services rendered and any legal recognition or encouragement, or legal restriction or regulation that tends to the advance and improvement of any profession or department of science, must of necessity, inure to the good and benefit of society at large for whose wants and needs the sciences and professions are cultivated and practiced. It is true, that in this movement, we propose to benefit directly all *competent* and *qualified dentists* in our State, to benefit dental surgery as a science and an honorable profession; and at the same time, and by the same means, to confer an equal and equivalent benefit upon society and our fellow men. In no just or fair sense can our proposed measure be denominated "class legislation."

We cannot be infringing, when we deny to the incompetent, the unscrupulous and the unprincipled, the privilege of undermining and sapping the foundations of our professional prosperity, of trifling with our health, of destroying our peace and comfort, and in some instances of sacrificing our lives. As reasonably might we talk of infringing the rights and privileges of the common swindler, the pick-pocket or the highway-man in the exercise of their vocations.

The only business or professional rights and privileges that will stand the crucial tests of reason, of justice, of law and of truth, are *those* rights in the employment and exercise of which we benefit ourselves, while doing a positive good and while conferring a relative, or equivalent benefit upon our neighbors and fellow men.

As dentists, we have the unquestionable right of supporting ourselves and our families; of acquiring wealth and reputation, by relieving the oral troubles and preserving the teeth of our patients, and patrons; but no one of us has the right to make money, or achieve honorable distinction by ignorantly aggravating the ills of our employers, or by abusing and destroying the invaluable organs we profess to restore and to preserve.

But, say some of our friends, "let these matters adjust and cure themselves; dont let us assume a suspicious attitude; dont let us *appear* to be seeking our own advantage, at the expense of others who claim as we do, to be dentists worthy of public confidence and patronage; let society purge and purify our profession by sustaining and encouraging the worthy, and reprobating and ignoring the worthless and mischievous."

Gentlemen, there are some ills and disorders in professions, in society, in morals and in physics, that left to themselves, can *never* reach a proper adjustment or radical cure. There are professional, as well as physical ulcers, and cancers, which *never* resolve or cure themselves, and it is no exaggeration or extravagance of language (as *you* well know,) for me to say, that *our* profession has been the subject and victim of these festering and cankerous sores sufficiently long. We have waited patiently for self correction, waited for the effect of social power and influence, and waited in vain. If there be merit in patience, we have surely earned it; if there be virtue in forbearance we have strained that principle, until it has lost its proverbial quality.

So long as we continue to wait for "self adjustment, or social regulation," just so long, may we expect to have our profession prostituted, and our escutcheon blurred and stained, the appreciation of our best skill and highest talent abated, and the recompense of our most finished and faithful service diminished by competition with pretenders and impostors.

In the larger towns and cities of the commonwealth, the centres of wealth and refinement, the evils of unrestricted quackery in our profession are less seriously felt than in the small towns and rural districts. In the cities, there are always found a wealthy and discriminating class of patrons and patients, who may be relied upon to appreciate and generously compensate skill and talent; but with the masses in the villages, hamlets and country districts, a dentist is a dentist; "good, bad, or indifferent," his placards and show-bills proclaim him a dentist, and the cheapness of his terms is the successful card he can always play against the most accomplished and deserving of our brotherhood.

I assert what I know to be a fact, that some of the most able, faithful, and skillful practitioners we have, are obscured, overslaughed, and kept poor and dependent, by the unlicensed competition of men who could not give you the technical names of the organs they assume to operate upon and preserve; who could not tell you, if their lives hung upon the answer, of what elements and substances the human teeth are composed, or even how and by what agencies the human mouth is opened or closed.

And yet, these men by reason of the unrestricted freedom accorded them here in Virginia, are monopolizing a large percentage of our practice which they command by underbidding and over-boasting, and which they make profitable to themselves, by the employment of the most inferior materials, and by the most hasty, slovenly and most discreditable of all imaginable botch-work. Thus has our profession in Virginia been abused and dishonored, and thus have some of our best men been kept down, impoverished, and in some instances completely bankrupted.

As President of this Association I have again and again been appealed to, from almost every section of the State, to do *something*; to inaugurate some measure to check, abate, or suppress the evils of indiscriminate and unlicensed dental practice, to do *something* to stay, or roll back the flood-tide of pretence and knavery which is washing away, sapping and undermining the very foundations of professional character and professional prosperity; which is blighting the hopes, blasting the prospects, and withering the energies of many of our capable, faithful, and deserving men.

The "rubber dentist," with his "Kit," his "Vulcanizer," his "Amalgam," and his "Gutta percha pellets," is ubiquitous; he springs out of the cross roads grocery, the shops and stores, the fields and back-woods forests. With the wonderful versatility and impudence that distinguishes these peripatetic vendors of rubber and cheap porcelain, they usually enhance their consideration, and increase their professional consequence, by the exhibition of varied talents and accomplishments. These gentry frequently combine with dental practice, the arts of cementing broken glass and crockery, the repairing of wood clocks and sewing machines, the refitting and furnishing of musical instruments, and such other gifts and talents as may be in demand, and serve to fill the intervals in more strictly professional (?) occupation.

I need not startle you, Gentlemen, when I tell you that I have had the honor of competing for practice, with an individual who tramped my neighborhood from house to house, and who received no mean amount of patronage, as he operated upon the teeth, the pianos, the sewing machines, the clocks and watches, the broken crockery and the dilapidated tin ware; and that that man tramp, impostor, and swindler as he was, enjoyed all the privileges and immunities that you, or I or any one else can claim under our present laws and usage.

As we have said, (and most of you too sadly know,) we have these disqualified, incompetent and mischievous men all over the State. Every community is more or less infested. We have them of home production, and of foreign importation. They are annual and perennial. When one vanishes, and disappears with his ill-gotten booty, followed by the imprecations and curses of his victims, another arises to take his place, and follow in his footsteps. And here Gentlemen, we fain would draw the veil and drop the curtain; but there is another element, another class in our profession, happily for us, not embraced in our Association, but scattered throughout the State, far more potent for mischief, far more effective in damaging and debasing our calling. These are men against whom no legislative enactments can prevail, men who have been instructed and qualified in our colleges, and who hold their diplomas, as evidence of merit and trustworthiness; and who have let themselves down, and drifted into the habits and practices, the measures and methods of the lowest and most contemptible of our common quacks and charlatans.

As 1 have said, such men cannot be reached or restrained by legal enactments. As a rule, they have been well taught, and could creditably acquit them-selves before any board of examiners, even if their diplomas did not except them from enquiry or examination. But Sirs, we can, and we do hold them up to professional reprobation; we can, and we do "pillory" them in professional indignation and contempt; we point to them as recreant sons of their "*Alma Mater*," and as the *black* and *scabby* sheep of our flock.

As I have the satisfaction of addressing one of the Professors of our Colleges, who has honored us with his presence, I beg leave to say, that I in no wise mean to reflect upon the Colleges, or the Faculties and teachers. I most cheerfully acquit them of all responsibility for the misdoings and short comings of some of their graduates. But I do mean to make this appeal to the Professors, that they spare no effort, while making good scholars and operators of their students, to make them also elevated gentlemen. Inspire them with sentiments of professional dignity and honor: teach them, that he who stains his diploma and by unworthy practices, is a bastard and an outcast, an alien and an enemy, that his rank is dishonored and forfeited, and that he no longer can claim a place in the "household of our faith."

And now Sirs, to return from this digression; we beg to submit that here in Virginia, we have tolerated these abuses in dental practice, about long enough. Thirty years ago, the State of Alabama found it necessary to protect her citizens, and her true professional men by the enactment of laws and provisions, regulating and restricting the practice ot dental surgery within her boundaries. Subsequently, and in succession, the States of New York, Ohio, Georgia, New Jersey, Pennsylvania, South Carolina, and we think several of the North Western States, enacted similar laws and regulations.

We are informed that the effect of this legislation has been wholesome and salutary wherever tried. It has protected society from imposition and abuse, it has protected the rights, and interests of the legitimate, and acknowledged members of our profession in those States. Dental science has been fostered and encouraged, and dental practice has been made more respectable and compensating.

But Sirs, one of the results of this neighboring legislation has been to Virginia, and other States having no law or regulations upon their subject, most prejudicial and unhappy, and deserves our most thoughtful consideration.

Virginia, and other States similarly circumstanced, have been made "lands of refuge," Penal Colonies, "Botany Bays," for such of those professional (?) gentlemen ? as were forced to "leave their country for their country's good"—and for such as could not at home practice their vocation with impunity or safety. My own distinguished (?) competitor, to whom I have made allusion, was an exile from the state of New York or Pennsylvania. There, he would have been promptly arrested, and fined or imprisoned. Here in Virginia, he could practice his frauds and impositions at his will and pleasure—"none dare molest, or make him afraid;" he was our peer and equal before Virginia law.

And now, Gentlemen, in view of the facts and considerations we have attempted to address to you, in view of the great injury we, as a profession, have so long and so patiently suffered, in view of the continued and increasing prostitution of a necessary and an honorable profession; and especially in view of the happy and healthy consequences and results which have been realized in our neighboring States, we feel that we cannot perform a more important or valuable service than to direct your thought and attention to this subject of legal regulation of the practice of dental surgery in our own commonwealth.

We trust it may be your pleasure to refer this subject to a committee of your members, with instructions to consider the policy and expediency of an appeal to the present legislature, for the enactment of such laws, regulating and restricting our "practice," as have been found favourable and efficient in other States and jurisdictions.

We submit in conclusion, that this proposed movement is timely and opportune. We believe that Virginia is upon the threshold of great and important changes; we believe that this grand old commonwealth, is about to emerge from the darkness and gloom that has shadowed and enveloped her varied and diversified interests;—that she is about to shake loose the shackles that have paralyzed her energies, diminished her productions, depressed her manufactures, crippled her trade, and obstructed her recuperation.

We believe that we are approaching an era in which all our civil and political rights and privileges are once more to be recognized and enjoyed, and all our material interests quickened and revived. Good government, wholesome laws, immigration, and the inflow of foreign capital, are to be mighty factors in this anticipated change. The arts, the sciences and the liberal professions will acknowledge and respond to the inspiration and impulse that returning prosperity always sets in motion; and we desire that department, *that profession* to which we have dedicated and devoted our labors and our lives, to come to the front disenthralled, unembarrassed, unobstructed, and with all the helps and aids of faithful *culture* and *practice* on *our* part, and all the assistance and encouragement that can be derived from wise and judicious legislation on the part of our representatives and lawgivers.

